# Influence of Cell Spreading Area on the Osteogenic Commitment and Phenotype Maintenance of Mesenchymal Stem Cells

**DOI:** 10.1038/s41598-019-43362-9

**Published:** 2019-05-03

**Authors:** Yingjun Yang, Xinlong Wang, Yongtao Wang, Xiaohong Hu, Naoki Kawazoe, Yingnan Yang, Guoping Chen

**Affiliations:** 1 Research Center for Functional Materials, National Institute for Materials Science, 1-1 Namiki, Tsukuba Ibaraki, 305-0044 Japan; 2 Department of Materials Science and Engineering, Graduate School of Pure and Applied Sciences, University of Tsukuba, 1-1-1 Tennodai, Tsukuba Ibaraki, 305-8577 Japan; 3 Graduate School of Life and Environmental Science, University of Tsukuba, 1-1-1 Tennodai, Tsukuba Ibaraki, 305-8571 Japan

**Keywords:** Biophysics, Mesenchymal stem cells

## Abstract

Osteogenic differentiation and commitment of mesenchymal stem cells (MSCs) is a complex process that is induced and regulated by various biological factors and biophysical cues. Although cell spreading area, as a biophysical cue, has been demonstrated to play a critical role in the regulation of osteogenic differentiation of MSCs, it is unclear how it affects the maintenance of the committed phenotype after osteogenic differentiation of MSCs. In this study, poly (vinyl alcohol) was micropatterned on a tissue culture polystyrene surface, and the micropatterns were used to culture MSCs to control their cell spreading area. The influence of cell spreading area on osteogenic differentiation and maintenance of the differentiated phenotype of MSCs was investigated. MSCs with a larger spreading area showed a higher degree of osteogenic differentiation, slower loss of differentiated phenotype and slower re-expression of stem cell markers compared with MSCs with a smaller spreading area. A large cell spreading area was beneficial for osteogenic differentiation of MSCs and maintenance of their differentiated phenotype.

## Introduction

Regeneration of deficient or injured tissues through tissue engineering and regenerative medicine approaches has been well developed for several decades^1^. Stem cells are considered attractive cell sources for these approaches. In particular, mesenchymal stem cells (MSCs), which possess self-renewability and multipotency to differentiate into osteoblasts^2^, smooth muscle cells^3–5^, chondrocytes^6–8^ and adipocytes^9–11^, have been demonstrated to be very useful cell sources^12–14^. The challenging issue for the utilization of stem cells for tissue engineering and regenerative medicine is how to control stem cell differentiation into desirable somatic cells and how to maintain the differentiated phenotype. Osteogenic differentiation and osteogenesis of MSCs have been broadly investigated using various osteogenic induction factors under different conditions.

Osteogenic induction factors, including dexamethasone, bone morphogenetic protein and transforming growth factor, have frequently been used for osteogenic differentiation and osteogenesis of MSCs. However, osteogenic differentiation and commitment of MSCs are extremely complicate processes that may be affected by various factors^15,16^. In addition to these biological induction factors, biophysical cues have also been reported to play an important role in stem cell differentiation^17,18^. A combination of biological factors and biophysical cues has been proposed to exhibit synergistic effects on the promotion of stem cell differentiation. Cell spreading area a typical biophysical cue and has been extensively studied for controlling stem cell functions. Cell spreading area has been reported to have regulative functions on cell proliferation^19^, migration^20–22^, differentiation^9^, transfection^23^ and reprogramming^24^. Cell morphology and spreading can be precisely controlled by using a variety of micropatterning techniques, such as microcontact printing, photolithography^25^ and stencil patterning^26^.

Although many studies have elucidated the influence of biophysical cues on stem cell differentiation, their influence on maintaining the differentiated phenotype of stem cells after removal of biological induction factors is unclear. Maintenance of differentiated states after stem cell differentiation is a critical issue for functional tissue regeneration because stem cell differentiation is a reversible process^27^, and it may be difficult to maintain the differentiated or committed phenotype after the removal of biological induction factors^28^. Determining the relationship between MSC morphology and maintenance of their differentiated phenotype is strongly anticipated. Therefore, in this study, micropatterned surfaces were prepared through ultraviolet (UV) -lithography and used for culturing MSCs to control their cell spreading area to investigate the influence of cell spreading area on osteogenic differentiation and maintenance of the osteogenically differentiated phenotype of MSCs.

## Results

### Preparation and characterization of micropatterned surfaces

**Figure 1:**
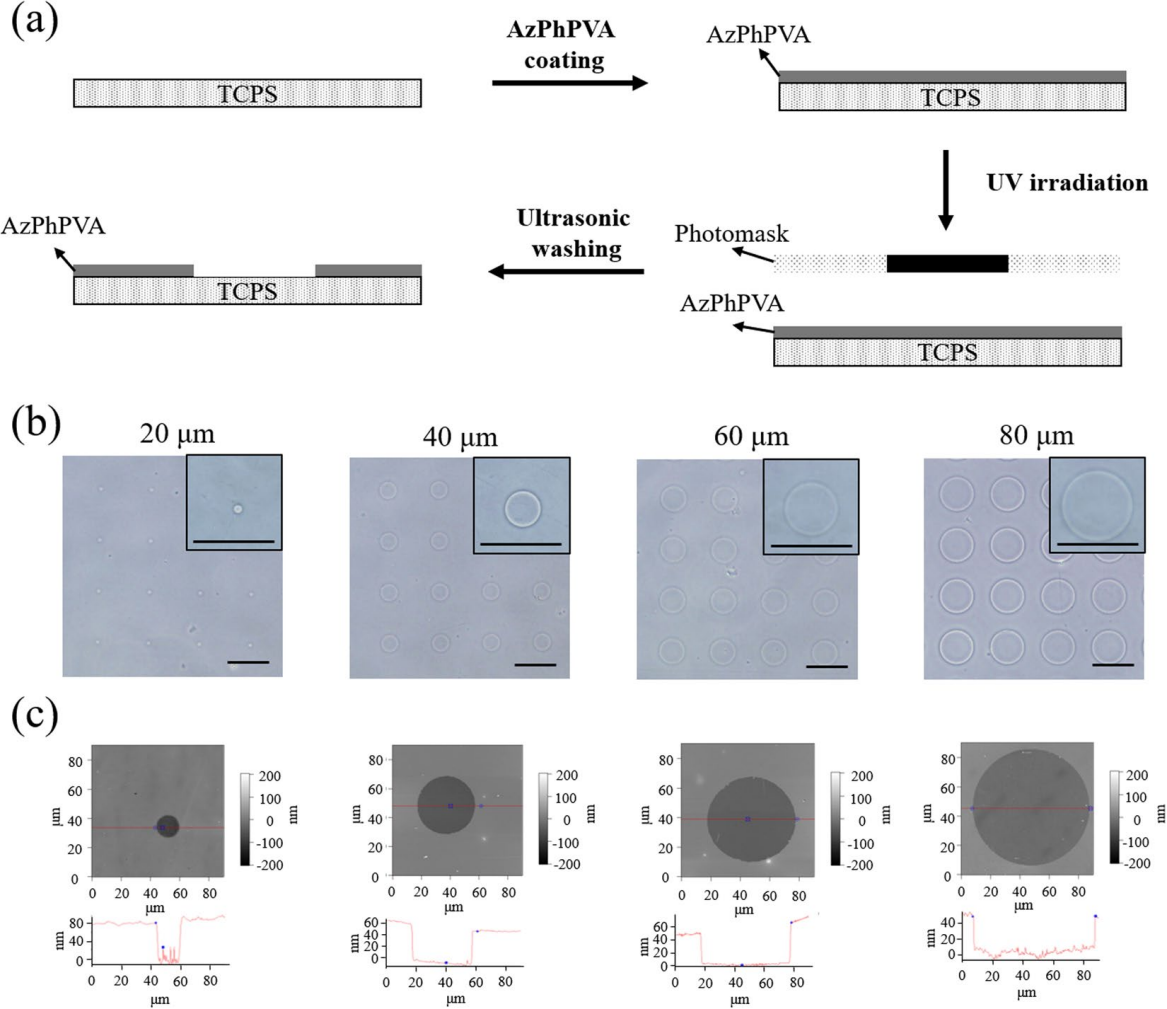
Preparation and characterization of PVA/TCPS micropatterned surfaces. (**a**) Preparation scheme of micropatterned surfaces by UV-lithography. (**b**) Representative photomicrographs of micropatterned surfaces. Scale bar: 100 μm. (**c**) Representative height images (up) and section images (down) of micropatterned surfaces characterized by AFM.

As shown at Fig. 1a, the poly (vinyl alcohol) (PVA)/tissue culture polystyrene (TCPS) micropatterned surfaces were prepared through UV-lithography. A thin layer of photo-reactive azidophenyl-derived poly (vinyl alcohol) (AzPhPVA) was firstly coated on cell-adhesive TCPS discs. The AzPhPVA-coated TCPS discs were UV irradiated through a transparent quartz photomask with non-transparent micropatterns. After UV irradiation, the photo-reactive AzPhPVA molecules below transparent regions of the photomask were grafted on the TCPS disc surface. The AzPhPVA molecules below non-transparent micropatterns of the photomask were easily stripped from the TCPS surfaces after the washing procedure. After washing, the micropatterned TCSP surfaces were obtained. Microdots of cell-adhesive TCPS were surrounded with a cell-repellent PVA layer. The circular micro-dot patterns prepared with a photomask having a micro-dot diameter of 20, 40, 60 and 80 μm and showed the same micropattern structure as that of the photomask (Fig. 1b,c). As characterized by AFM, the micropatterned micro-dots had a diameter of 20.2 ± 0.5, 41.3 ± 0.4, 60.7 ± 1.4 and 82.1 ± 1.4 μm, respectively. The area of each type of circular micropatterns was 321.5 ± 17.2, 1338.6 ± 23.0, 2894.0 ± 134.9 and 5294.4 ± 184.7 μm^2^_,_ respectively. The depth of PVA layer in the four types of micro-dot patterns was 64.6 ± 3.1, 58.4 ± 3.4, 58.3 ± 3.2 and 60.6 ± 4.9 nm, respectively. All the micro-dot patterns had almost the same thickness of the surrounding PVA layer, which could protect protein adhesion and protect cell adhesion. Cells could only adhere on the TCPS micro-dots to form the micropatterned cells.

### Cell morphology and actin filaments structure

**Figure 2:**
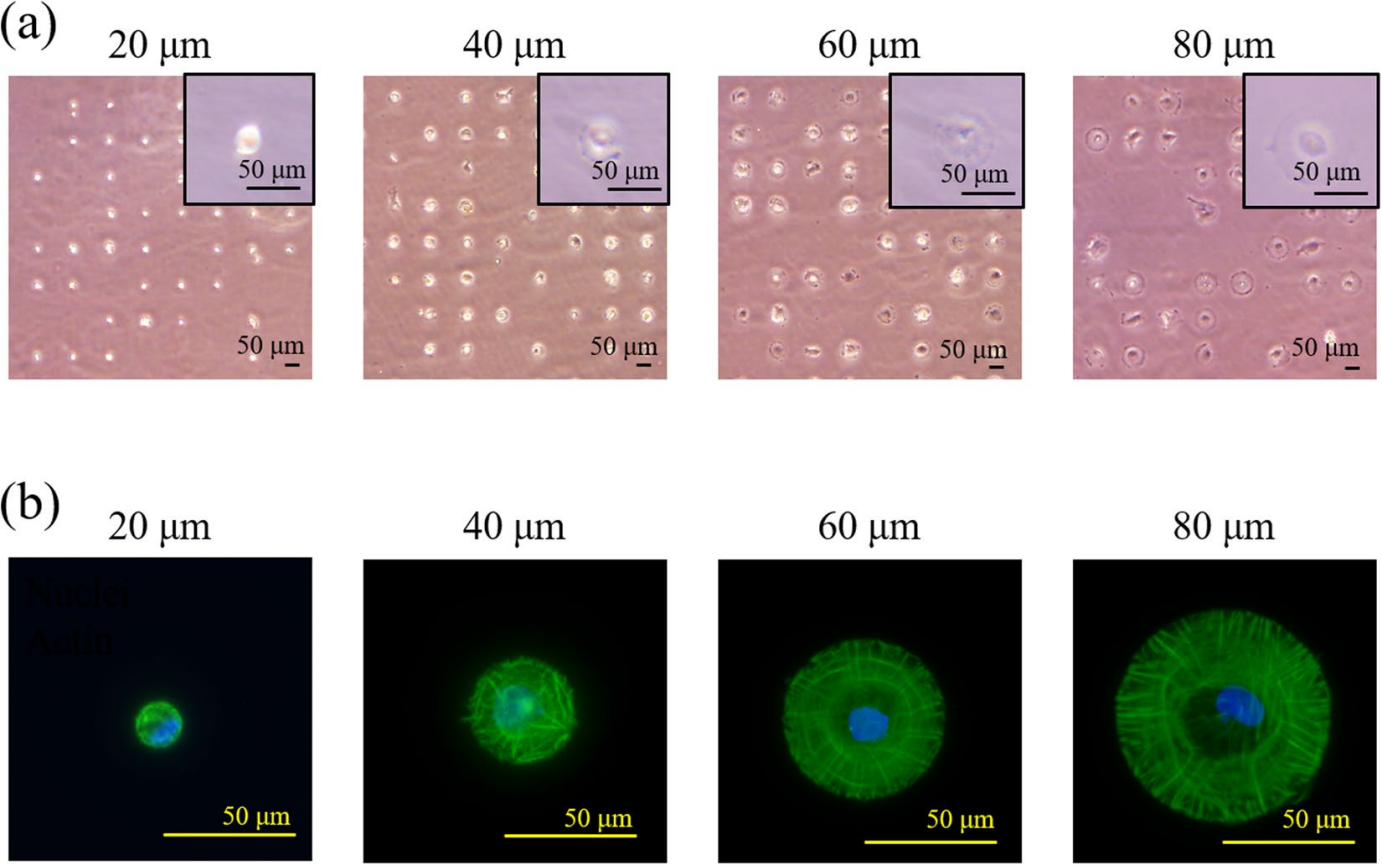
Cell adhesion and morphology on micropatterned surfaces. (**a**) Representative photomicrographs of MSCs adhered on micropatterned surfaces after culturing in basal medium for 1 day. (**b**) Representative images of actin (green)- and nuclei (blue)-stained images of MSCs, as shown in (**a**).

PVA/TCPS circular micropatterns were used for culture of MSCs to control cell spreading area. After culture in MSCGM^TM^ growth medium (basal medium) for 24 hours, MSCs adhered on the TCPS micro-dots of the micropatterned surfaces not on the PVA-grafted regions. At the seeding density of 3000 cells/cm^2^, most of the cells showed single cell adhesion on each TCPS micro-dots of the micropatterned surfaces (Fig. 2a). MSCs on the small TCPS micro-dots with a diameter of 20 μm did not spread, while MSCs on the large TCPS micro-dots with diameters of 40, 60 and 80 μm spread along the micro-dots. The cells showed the same circular morphology as that of the micro-dots. Cell spreading area was almost the same as that of the micro-dots. Therefore, cell morphology and spreading area were precisely controlled by the micropatterned surfaces. Staining of actin filaments and nuclei revealed that actin filament structure was different when the micro-dot size was changed (Fig. 2b). The actin filaments of micropatterned MSCs on the micro-dots with a diameter of 20 μm showed random structure without clear alignment. When the spreading area of micropatterned MSCs increased, alignment of actin filaments became more evident. The micropatterned MSCs on the micro-dots with a diameter of 80 μm exhibited highly aligned actin filaments that were well assembled along the radial and concentric directions.

### Influence of cell spreading area on CD105 expression

**Figure 3:**
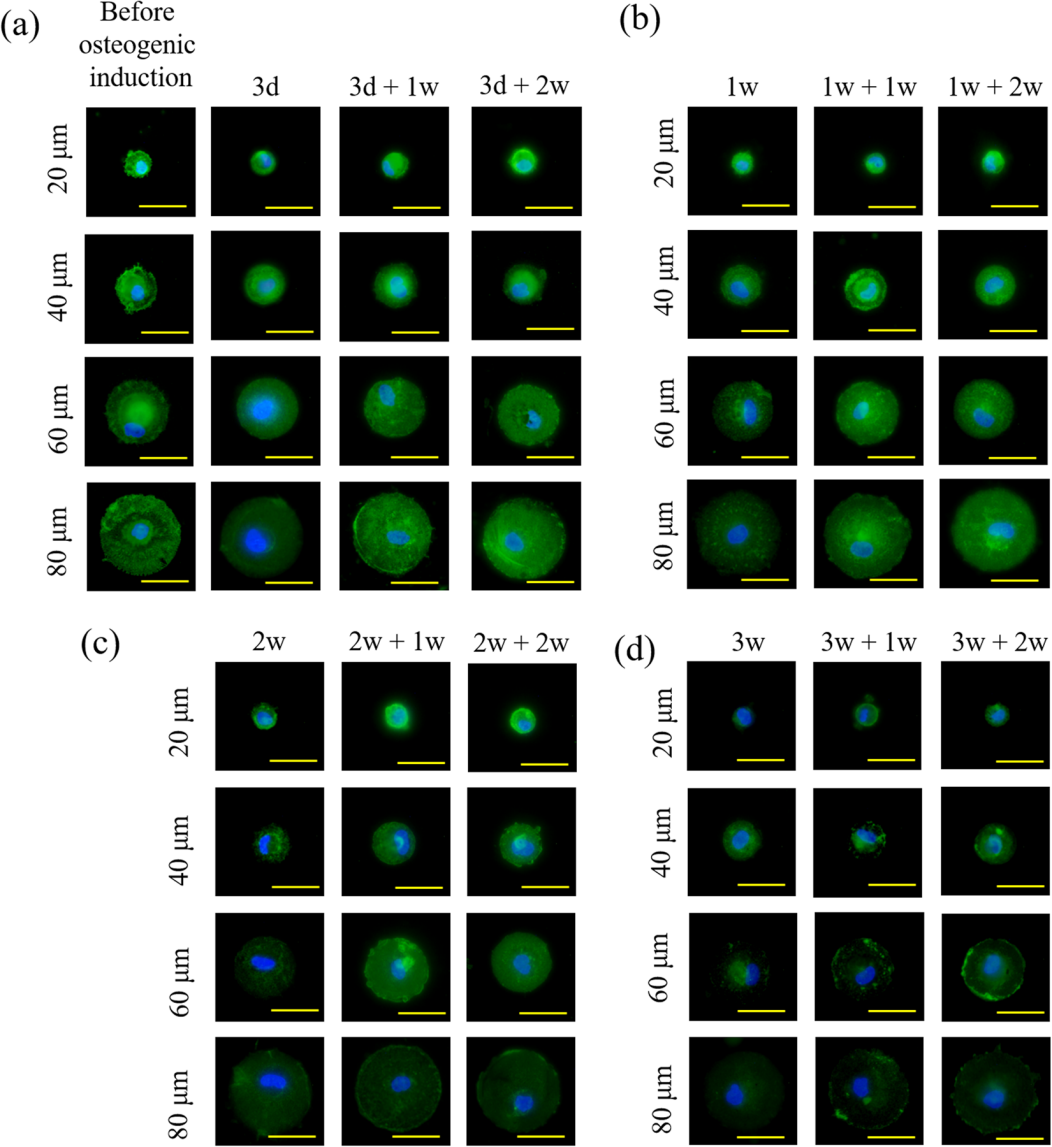
Representative images of CD105 immunofluorescence staining of (**a**) micropatterned MSCs cultured in osteogenic induction medium for 3 days (3 d) and re-cultured in basal medium for 1 (3 d + 1 w) and 2 (3 d + 2 w) weeks; (**b**) micropatterned MSCs cultured in osteogenic induction medium for 1 week (1 w) and re-cultured in basal medium for 1 (1 w + 1 w) and 2 (1 w + 2 w) weeks; (**c**) micropatterned MSCs cultured in osteogenic induction medium for 2 weeks (2 w) and re-cultured in basal medium for 1 (2 w + 1 w) and 2 (2 w + 2 w) weeks. (**d**) Micropatterned MSCs cultured in osteogenic induction medium for 3 weeks (3 w) and re-cultured in basal medium for 1 (1 w + 1 w) and 2 (1 w + 2 w) weeks. Scale bar: 50 μm.

CD105 is a representative surface marker of MSCs. CD105 expression was analysed to evaluate the stemness of MSCs. The micropatterned MSCs cultured at different conditions were immunofluorescently stained with a CD105 antibody. The fluorescent intensity of each micropatterned MSCs was calculated to indicate the CD105 expression level. Representative images of immunofluorescence staining of CD105 of MSCs cultured in basal medium before osteogenic induction; after culture in osteogenic induction medium for 3 days or 1, 2 and 3 weeks (osteogenic induction); and after culture in osteogenic induction medium for 3 days or 1, 2 and 3 weeks followed by culture in basal medium for 1 and 2 weeks (re-culture in basal medium after initial osteogenic induction) are shown in Fig. 3. Approximately all of the micropatterned MSCs showed positive staining of CD105 when they were cultured in basal medium before osteogenic induction culture. When MSCs were cultured in osteogenic induction medium, the fluorescence of CD105 staining became weak. When the culture medium was changed from osteogenic induction medium to basal medium after initial osteogenic induction culture for a certain period, the fluorescence intensity of stained CD105 increased again.

**Figure 4:**
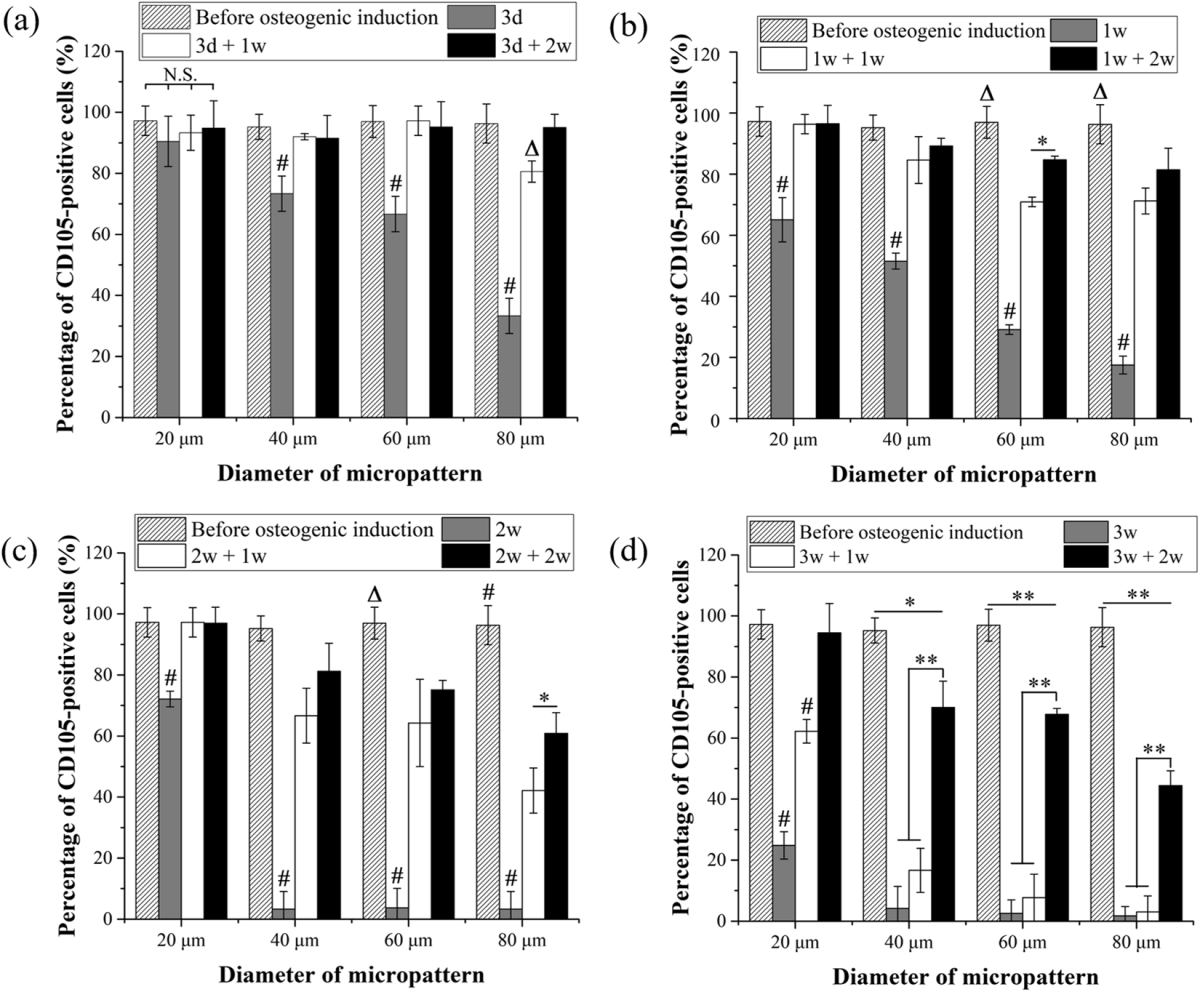
Percentage of micropatterned MSCs that were positively stained with a CD105 antibody. The cell culture conditions were the same as in Fig. 3. Data are presented as the means ± SDs, n = 3, *p < 0.01, **p < 0.001. # or ∆ represents *p* < 0.01 or *p* < 0.001 compared with the other three groups of the same cell spreading area but different cell culture conditions.

CD105-positive MSCs were counted from the immunofluorescent staining of CD105, and the percentage of CD105-positive MSCs was calculated to show CD105 expression levels (Fig. 4). During osteogenic induction culture, CD105 expression levels of MSCs decreased with the increase in cell spreading area and induction culture time. Although CD105 expression of MSCs with the lowest spreading area did not change during the first 3 days of induction culture, CD expression levels of MSCs cultured at all other conditions significantly decreased. In particular, MSCs with large spreading area (on the micro-dots having a diameter of 40, 60 and 80 μm) expressed very low levels of CD105 after 2 and 3 weeks of induction culture. MSCs with the largest spreading area showed the lowest level of CD105 expression after induction culture.

When the initially induced MSCs were re-cultured in basal medium, CD105 expression levels showed some rebound. The rebound degree was dependent on cell spreading area, initial induction culture time and re-culture time in basal medium. The rebound level of CD105 was high when the cell spreading area was small, initial induction culture time was short and re-culture time was long, and vice versa. CD105 expression in MSCs having the lowest spreading area could completely rebound to the original level for all the re-culture conditions except the culture condition of 3 d + 1 w. On the other hand, CD105 expression in MSCs having the largest spreading area could not rebound to the original level for either re-culture condition except the culture condition of 3 d + 2 w. The rebound level of CD105 was the lowest when MSCs with the largest spreading area were cultured at the condition of 3 d + 1 w.

### Influence of cell spreading area on ALP activity and calcium deposition

**Figure 5:**
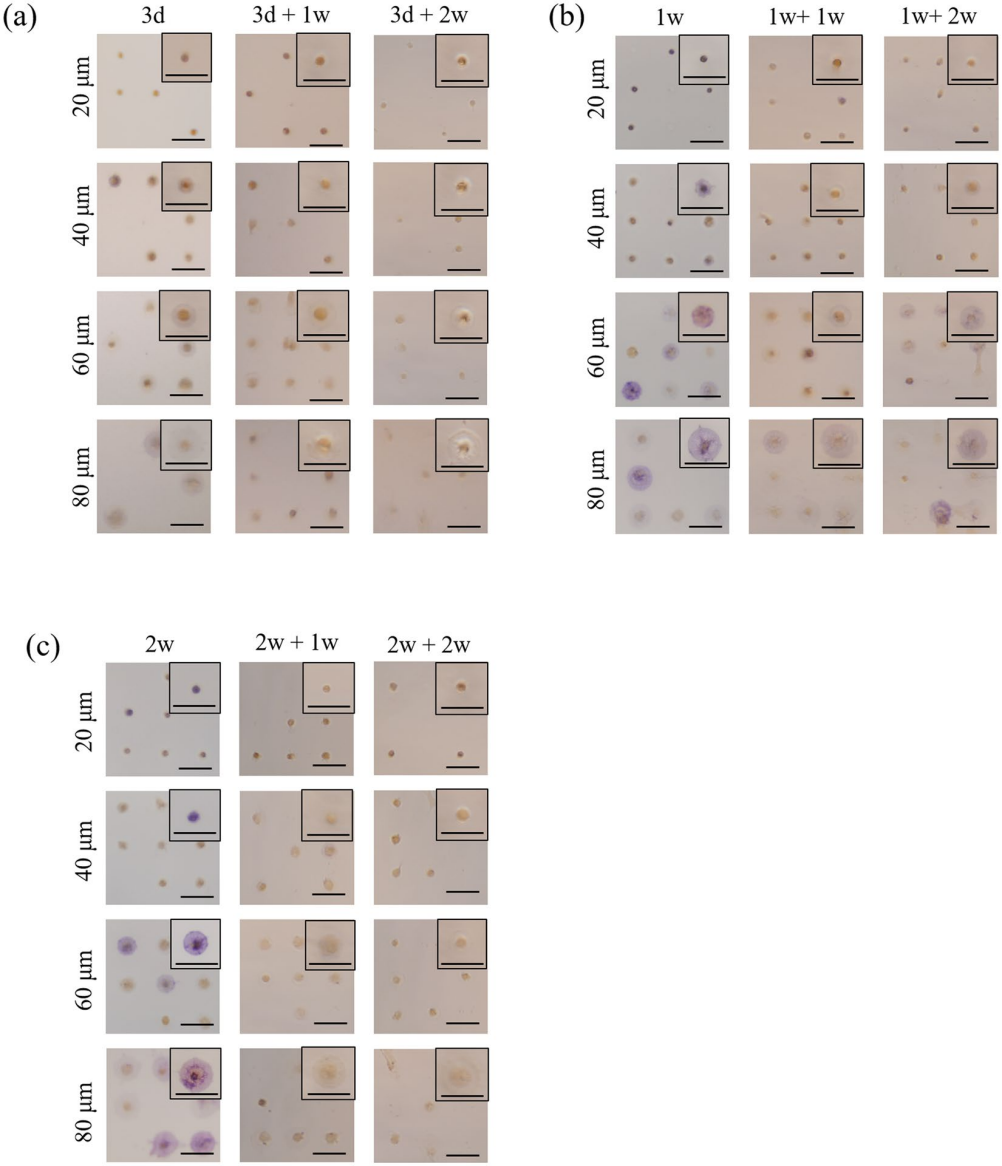
Representative images of ALP staining of micropatterned MSCs. Positively stained cells are purple, and negatively stained cells are brown. The cell culture conditions were the same as in Fig. 3. Scale bar: 100 μm.

Alkaline phosphate (ALP) is an early marker of osteogenic differentiation of MSCs. ALP staining of micropatterned MSCs was conducted to analyse the early osteogenic differentiation after the cells were cultured in osteogenic induction medium for 3 days or 1 and 2 weeks (osteogenic induction) and after the cells were cultured in osteogenic induction medium for 3 days or 1 and 2 weeks followed with re-culture in basal medium for 1 and 2 weeks. The positively stained cells appeared in purple, and negatively stained cells appeared in brown (Fig. 5). ALP staining was not evident when cell spreading area was small and osteogenic induction culture time was short. ALP staining became evident when the cell spreading area was large and induction time was long.

**Figure 6:**
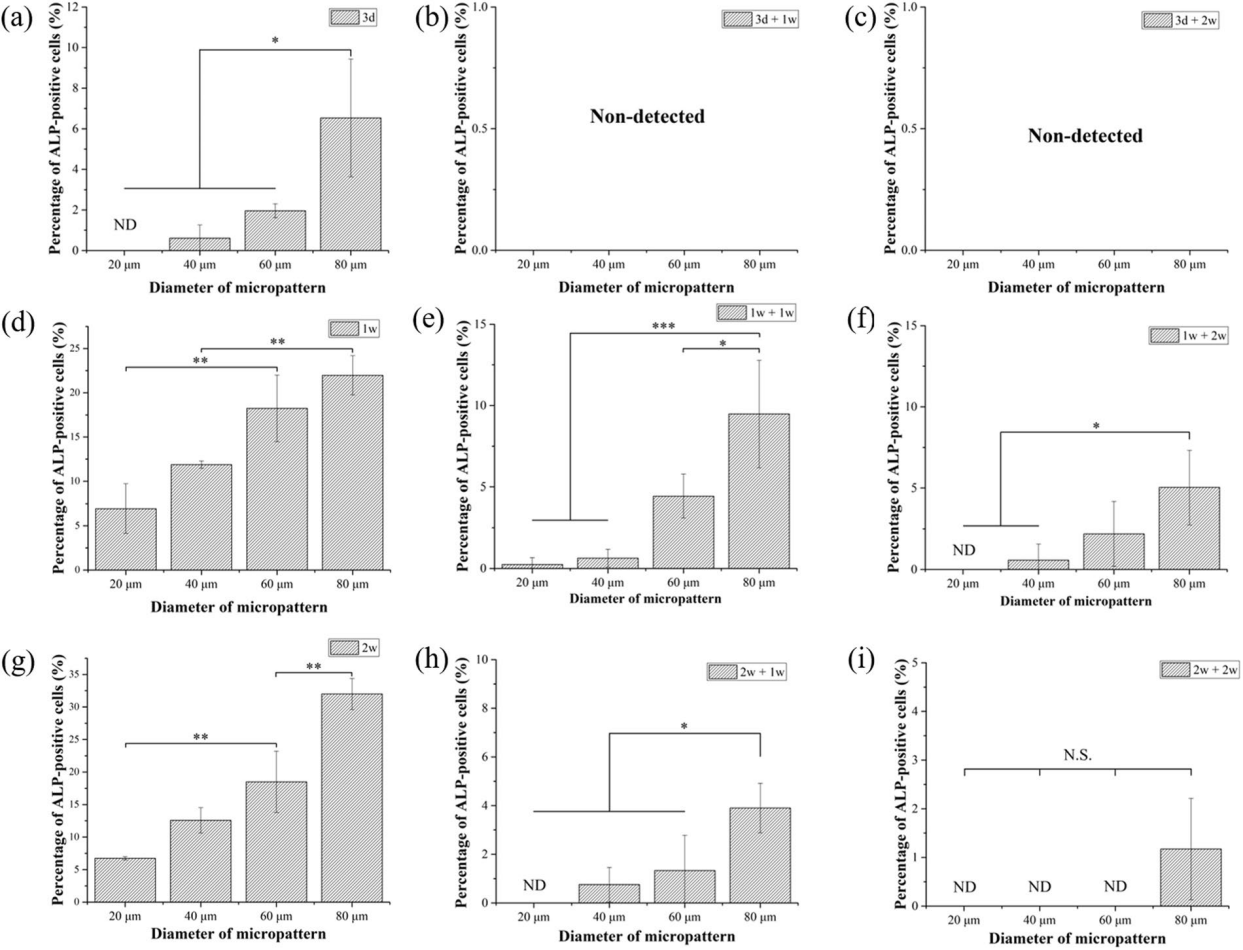
Percentage of ALP-positive micropatterned MSCs. The cell culture conditions were the same as in Fig. 3. Data are presented as the means ± SDs, n = 3, *p < 0.05, **p < 0.01, ***p < 0.001, ND: non-detected.

Percentage of cells that were positively stained with ALP was calculated from the images of ALP staining (Fig. 6). ALP activity of the micropatterned MSCs increased when the spreading area of MSCs and the induction culture time increased. For all the induction culture times, MSCs with the largest spreading area showed the highest percentage of ALP-positive cells, while MSCs with the lowest spreading area showed the lowest percentage. When the culture medium was changed from induction one to basal one, ALP-positive cells decreased significantly for all the micropatterned cells. The percentage of ALP-positive cells also decreased when the re-culture time was extended. When the initial induction time was short (3 d), the percentage of ALP-positive cells decreased to zero. Some cells still expressed ALP even after re-culture in basal medium if the initial induction culture was long (1 w and 2 w).

**Figure 7:**
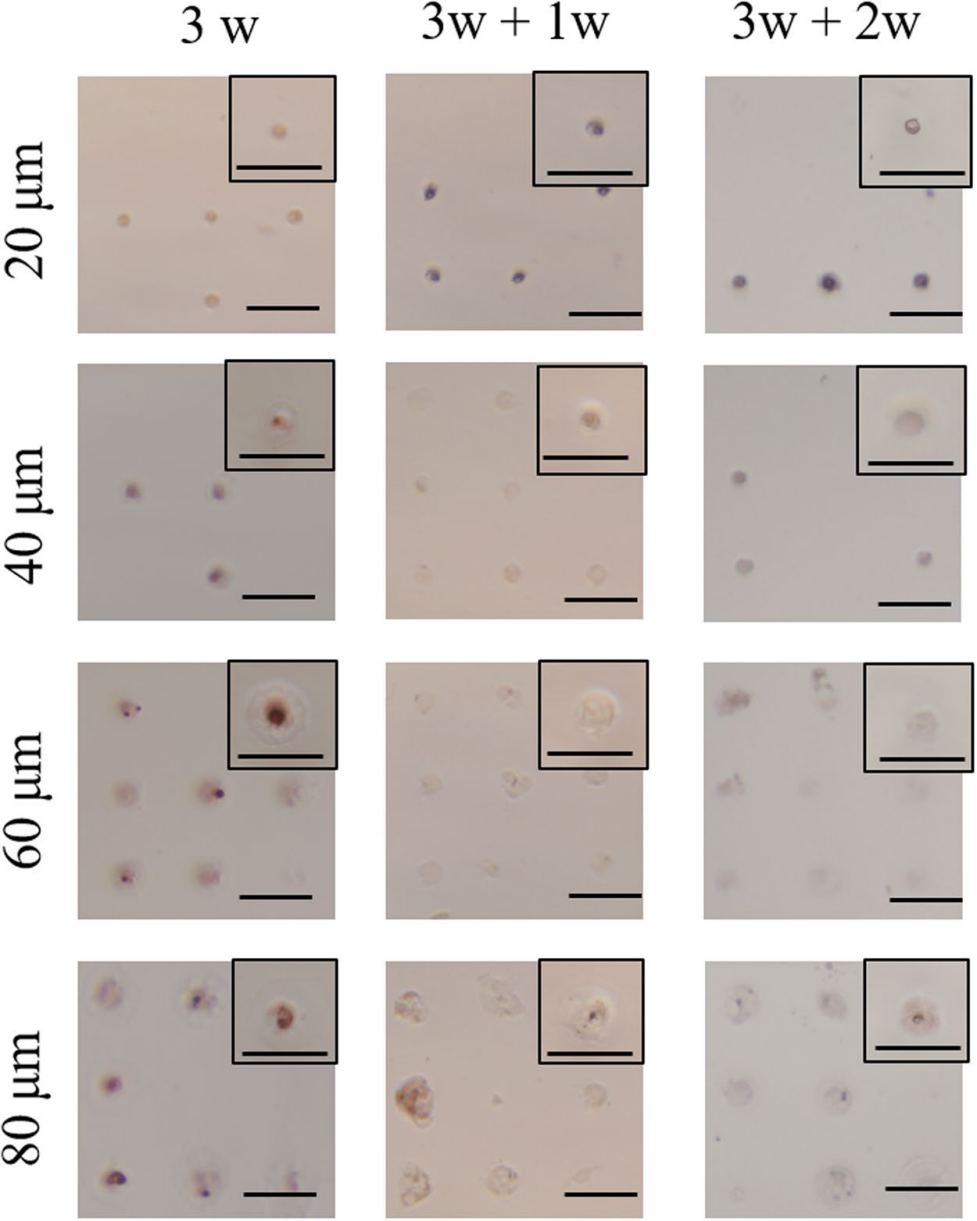
Representative images of ARS staining of micropatterned MSCs. ARS-positive MSCs appeared as red spots, and ARS-negative cells are brown. The cell culture conditions were the same as in Fig. 3. Scale bar: 100 μm.

**Figure 8:**
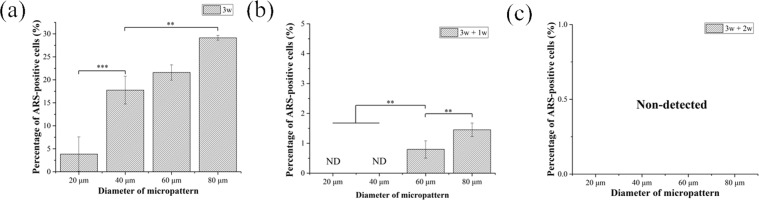
Percentage of ARS-positive micropatterned MSCs. The cell culture conditions were the same as in Fig. 3. Data are presented as the means ± SDs, n = 3, **p < 0.01, ***p < 0.001, ND: non-detected.

Calcium deposition is a late marker of osteogenic differentiation. Calcium deposition of micropatterned MSCs after osteogenic induction medium for 3 weeks followed with or without re-culture in basal medium for 1 and 2 weeks was stained by ARS to further investigate their osteogenic differentiation. ARS-positive MSCs appeared as red spots, and ARS-negative cells appeared in brown (Fig. 7). Calcium deposition was not or very weakly detected when osteogenic induction culture time was short (3 days, 1 and 2 weeks). Only after osteogenic induction culture for 3 weeks, calcium deposition was obviously detected. More cells showed calcium deposition when the cell spreading area became larger. The percentage of ARS-positive cells was calculated from the images of ARS staining (Fig. 8). After osteogenic induction culture for 3 weeks, the percentage of ARS-positive cells increased with cell spreading area. MSCs with the largest spreading area had the highest Ca deposition, while MSCs having the lowest spreading area showed the lowest Ca deposition. When the initially induced MSCs were re-cultured in basal media, the percentage of ARS-positive cells decreased significantly. The percentage of ARS-positive cells further decreased as the time of re-culture in basal medium increased. After re-culture in basal medium for 2 weeks, no ARS-positive cells were detected.

## Discussion

Since osteogenically differentiated MSCs may lose their differentiated phenotype after withdrawing the biochemical induction factor, osteogenesis of MSCs is regarded as a reversible process^29^. Therefore, some release systems have been designed for a sustained supply of induction factors for a long period to maintain the differentiated phenotype^30,31^. Meanwhile, cell morphology, including cell spreading and elongation, affects osteogenic differentiation of MSCs^32,33^. However, it is unclear whether cell morphology has any impact on the maintenance of differentiated phenotype after osteogenic differentiation of MSCs. Therefore, in this study, PVA/TCPS micro-dots patterns with different diameters were prepared used for culture of MSCs to control their cell spreading area for investigation of the influence of cell spreading area on osteogenic commitment and maintenance of the differentiated phenotype of MSCs. When MSCs were cultured on the PVA/TCPS micropatterned surfaces, the spreading area of MSCs was precisely controlled, and cell spreading area was determined by the area of TCPS micro-dots. The well-aligned and organized actin filaments presented in large MSCs indicated that actin polymerization was promoted by cell spreading (Fig. 2b). Well organized actin filaments can lead to high cell contractility^34^, which is critical in regulation of osteogenic differentiation^33,35^ and stemness maintenance^36^ of MSCs.

Osteogenic differentiation of MSCs after osteogenic induction culture, variation of differentiated phenotype after re-culture in basal medium and stem cell marker expression were evaluated by staining stem cell and osteogenic differentiation markers. CD105 is a surface marker to identify mesenchymal stem cells^37^ and evaluate their multipotency^38^. CD105 expression on the membrane surface of MSCs is associated with the process of osteogenesis^39–41^. Based on previous studies, CD105 expression decreases during osteogenesis of MSCs. In this study, CD105 expression was used to evaluate the stemness of micropatterned MSCs during initial osteogenic induction culture and re-culture in basal media. ALP and calcium deposition, which are respective early and late markers of osteogenic differentiation of MSCs, were used to evaluate osteogenic differentiation.

CD105 expression in the micropatterned MSCs decreased as the osteogenic induction period was extended. In each group, MSCs with high spreading area showed low CD105 expression levels and high ALP activity and calcium deposition after induction culture. As previously reported, large MSCs can promote osteogenesis^42^; thus, the high level of CD105 expression by low spreading area MSCs in this study is attributed to the inhibition of osteogenesis. The result was also consistent with the previous result that multipotency of MSCs decreases during the osteogenesis process^41^, and small spreading area is beneficial for maintenance of stemness after a long culture period in basal medium^36^.

After the osteogenic induction, the medium was replaced by basal medium. The osteogenesis of micropatterned MSCs was inhibited, and CD105 expression was partially or fully recovered. Combined with the previous study showing that the dedifferentiated MSCs obtained multipotency again after withdrawal of the biochemical induction factors^29^, recoverability of stemness was related with cell spreading area and the osteogenic induction period. Completely recovered CD105 expression was only observed in small MSCs or MSCs that were osteogenically induced for a short period. The results indicated that stemness recoverability could be compromised by the promotion of osteogenesis, or stemness could only be recovered at the initial stage of osteogenesis. Cell spreading area not only affects osteogenic differentiation of MSCs but also affected de-differentiation of the initially differentiated cells and recovered expression of stem cell markers. Large MSCs facilitated osteogenic differentiation of MSCs more strongly than small MSCs, while small MSCs could return to the stem cell stage more easily than large cells. The recovered MSCs should have the same osteogenic differentiation capacity as the initial MSCs^29^. Human bone marrow-derived MSCs were used, and the influence of spreading area on osteogenic differentiation was investigated in this study. The results may be applicable to other types of MSCs, such as human adipose-derived MSCs and umbilical cord-derived MSCs. Cell spreading area may also affect the maintenance of the differentiated phenotype of MSCs after differentiation of other lineages, such as adipogenic or chondrogenic differentiation, although further study is needed for confirmation.

In summary, using UV-lithography and photoreactive AzPhPVA, PVA/TCPS micropatterned surfaces were prepared and used to control the spreading area of MSCs to investigate the influence of cell morphological cues on osteogenic differentiation and commitment of MSCs. Large MSCs promoted osteogenic differentiation but suppressed the recoverability of stem cell marker expression. By contrast, small MSCs inhibited osteogenic differentiation but facilitated the recovery of stem cell marker expression. The results indicate that cell spreading area is an important factor for stem cell differentiation and commitment.

## Methods

### Preparation and characterization of micropatterns

Photo-reactive AzPhPVA was used to prepare PVA micropatterns on TCPS discs through photo-lithography. AzPhPVA was synthesized by introducing azidophenyl groups in poly (vinyl alcohol) as previously reported^43^. TCPS discs (2.5 × 2.5 cm) were cut from Falcon^TM^ tissue culture treated flasks. Briefly, 0.2 mL of 0.3 mg/mL AzPhPVA aqueous solution was dropped on the central area (1 × 1 cm) of each TCPS disc. After the AzPhPVA aqueous solution was air-dried at room temperature in the dark, the AzPhPVA-coated TCPS discs were irradiated by ultraviolet light (UV, Funa-UV-linker FS-1500, 0.25 J/cm^2^) through a micropatterned photomask. The photomask was a quartz wafer containing non-transparent micro-dots with diameters of 20, 40, 60 and 80 μm. After UV irradiation, the un-reacted AzPhPVA molecules below non-transparent micro-dots of the photomask were completely removed after ultrasonic washing in Milli-Q water. The micropatterned discs were sterilized by immersing them in 70% ethanol aqueous solution for 20 min and rinsed by aseptic Milli-Q water before cell culture.

The micropatterned surfaces were observed using a phase-contrast microscope (BX51, Olympus, Tokyo, Japan) and characterized by an MFP-3D-BIO atomic force microscope (AFM, Asylum Research Corporation, Santa Barbara, CA). For AFM characterization, a cantilever (spring constant: 0.06 N/m; oscillation frequency: 12–24 kHz; DNP-10, Bruker) with a nitride tip was used. Contact mode in Milli-Q water was performed during the scanning process.

### Cell culture

Human bone marrow-derived mesenchymal stem cells (MSCs) were purchased from Lonza Walkersville, Inc. at passage 2. A cell colony from a single MSCs was used for the following cell culture experiments. A cell colony of MSCs was obtained using a previously reported method^36^. Briefly, less than 30 cells were seeded onto a cell culture dish (*d* = 10 cm) and cultured in MSCGM^TM^ growth medium (Lonza Group Ltd., supplied with 10% serum, 2% L-glutamine and 0.1% gentamicin sulfate amphotericin b) for 3 weeks to obtain cell colonies. Cell colonies with a diameter greater than 4 mm were collected and sub-cultured in 25-cm^2^ TCPS flasks for an additional 3 weeks to obtain the homogeneous cell mass. The purified MSCs at passage 4 were used for following experiments. The sterilized micropatterned discs were placed in 6-well TCPS plates, and 3 mL MSCGM^TM^ medium was added per well. Glass rings with a 1.5-cm inner diameter were placed on each micropatterned disc to constrain the seeded cells on the micropatterns. Then, 200 μL cell suspension solution (2.7 × 10^4^ cells/mL) was added within each glass ring (3000 cells/cm^2^), which had been optimized to allow single cell attachment on each micro-dot. Glass rings were removed after the cells were cultured on the micropatterned surfaces in a humidified incubator at 37 °C and 5% CO_2_ for 6 hours. After another 18 hours of culture, cell attachment on the micropatterned surfaces was observed using a phase-contrast microscope.

After MSCs were cultured on the micropatterned surfaces in MSCGM^TM^ medium (basal medium) for 24 hours, the culture medium was replaced by osteogenic induction medium (DMEM medium supplied with 10% foetal bovine serum, 1000 mg/L glucose, 584 mg/L glutamine, 100 U/mL penicillin, 100 μg/mL streptomycin, 0.1 mM nonessential amino acids, 50 mg/L ascorbic acid, 100 nM dexamethasone and 10 mM β-glycerophosphate disodium salt hydrate). The osteogenic induction culture was continued for 3 days or 1, 2 and 3 weeks. To investigate whether osteogenically differentiated cells could maintain their differentiated phenotype, the osteogenic induction medium was changed to basal medium without osteogenic induction factors. After osteogenic induction culture for 3 days or 1, 2 or 3 weeks, the osteogenic induction medium was replaced with basal medium, and the cells were further cultured for 1 or 2 weeks under 37 °C and 5% CO_2_ in a humidified incubator. The medium was refreshed every 3 days.

### Fluorescence staining of actin and nuclei

After MSCs were cultured on the micropatterned surfaces in MSCGM^TM^ medium for 24 hours, the cells were fixed by 4% cold paraformaldehyde aqueous solution for 10 minutes. The fixed cells were permeabilized by 1% Triton^TM^ X-100 for 2 minutes and blocked by immersing in 1% bovine serum albumin (BSA) aqueous solution for 30 minutes in room temperature. The cells were stained by incubation with Alexa Fluor-488^®^ phalloidin in room temperature in the dark for 20 minutes. After being washed with PBS solution thrice, the samples were dried at room temperature in the dark and mounted with VECTASHEILD^®^ (with DAPI, Vector Laboratories, Inc.). Fluorescence images of each sample were captured through an Olympus BX51 microscope (Olympus, Tokyo, Japan).

### Immunofluorescence staining of stem cell marker

A representative surface marker of MSCs, CD105 (endoglin), was stained after MSCs were cultured on the micropatterned surfaces at the above-mentioned conditions. Samples were fixed by 4% cold paraformaldehyde aqueous solution for 10 minutes and blocked by 1% BSA and 0.3 M glycine for 30 minutes in room temperature. Samples were incubated with a primary CD105 antibody (Invitrogen, CA, USA) aqueous solution diluted at 1:500 in 1% BSA for 1.5 hours in room temperature. After three rinses in PBS, the samples were incubated with an Alexa Fluor-488^®^ conjugated goat anti-mouse IgG antibody (Invitrogen, CA, USA) at a dilution ratio of 1:1000 in PBS at room temperature for 1 hour. After three rinses in PBS and drying at room temperature in the dark, the samples were mounted by VECTASHEILD^®^ (with DAPI, Vector Laboratories, Inc.). The fluorescent images of stained cells were obtained through a fluorescence Olympus BX51 microscope at a fixed parameter (5 s, ISO:200). The corrected total fluorescence (*CTF*_*Cell*_) of CD105 was calculated using ImageJ software (National Institutes of Health, Bethesda, Maryland, USA). The area (*A*_*Cell*_) and integrated intensity (*I*_*Cell*_) of each micropatterned cells were measured. The area (*A*_*Background*_) and integrated intensity (*I*_*Background*_) of micropatterns without cells were also measured and set as background. The *CTF* of CD105 in micropatterned cells was calculated as *CTF*_*Cell*_ = (*I*_*Cell*_/*A*_*Cell*_ − *I*_*Background*_/*A*_*Background*_) × *A*_*Cell*_. The *CTF* of micropatterned cells, which were only incubated with secondary antibody (Alexa Fluor-488^®^ conjugated goat anti-mouse IgG antibody) without incubation with first antibody, were calculated and set as a control group (*CTF*_*Control*_). CD105-positive cells were defined as the cells having 50 times higher fluorescence intensity than the control group (*CTF*_*Cell*_/*CTF*_*Control*_ > 50). The ratio of CD105-positive cell number to the total cell number was calculated to evaluate the stemness of MSCs. Greater than 150 cells from 3 independent experiments were used for the analysis. Each sample was numbered and blinded during analysis.

### Alkaline phosphatase and alizarin red S staining

Osteogenic differentiation of MSCs on the micropatterned surfaces during osteogenic induction culture was evaluated by alkaline phosphatase (ALP) staining and alizarin red S (ARS) staining. After MSCs were culture on the micropatterned surfaces for a designated time, the cells were rinsed with pre-warmed PBS solution twice and fixed with 4% cold paraformaldehyde aqueous solution for 10 minutes. After thrice washes in PBS, the fixed cells were immersed in staining solution of ALP or ARS at room temperature for 10 minutes. ALP staining solution was composed of 56 mM 2-amino-2-methyl-1,3-propanediol (pH = 9.9, Sigma-Aldrich Co. LLC., USA), 0.1 wt% naphthol AS-MX phosphate (Sigma-Aldrich Co. LLC, USA) and 0.1 wt% Fast Blue RR salt (Sigma-Aldrich Co. LLC., USA). ARS staining solution was 0.1% ARS solution. Optical images of the stained cells were obtained through a phase-contrast microscope. The optical images were analysed by Colour Deconvolution plugin of ImageJ to discriminate ALP-positive and -negative cells. In the original optical images, colour-specific vectors were assigned as purple and brown channels. The percentage of ALP- or ARS-positively stained cells was calculated. Greater than 150 cells from 3 independent micropattern discs were used for the analysis. Each sample was numbered and blinded during analysis.

### Statistical analysis

Statistical analysis was performed using a one-way analysis of variance (ANOVA) with Tukey’s post hoc test for multiple comparisons to confirm the significant differences among samples. The data are presented as the means ± standard deviations (SDs). Groups were considered to be significantly different when *p* < 0.01.
